# Infection Profiles of Selected Aquabirnavirus Isolates in CHSE Cells

**DOI:** 10.1371/journal.pone.0134173

**Published:** 2015-08-11

**Authors:** Amr A. A. Gamil, Øystein Evensen, Stephen Mutoloki

**Affiliations:** Department of Basic Sciences and Aquatic Medicine, Faculty of Veterinary Medicine and Biosciences, Norwegian University of Life Sciences, P.O. Box 8146, Dep. 0033 Oslo, Norway; CEA, FRANCE

## Abstract

The wide host range and antigenic diversity of aquabirnaviruses are reflected by the presence of a collection of isolates with different sero- and genotypic properties that have previously been classified as such. Differences in cytopathogenic mechanisms and host responses induced by these isolates have not been previously examined. In the present study, we investigated infection profiles induced by genetically and serologically closely related as well as distant isolates *in-vitro*. CHSE-214 cells were infected with either E1S (serotype A3, genogroup 3), VR-299 (serotype A1, genogroup 1), highly virulent Sp (TA) or avirulent Sp (PT) (serotype A2, genogroup 5). The experiments were performed at temperatures most optimum for each of the isolates namely 15°C for VR-299, TA and PT strains and 20°C for E1S. Differences in virus loads and ability to induce cytopathic effect, inhibition of protein synthesis, apoptosis, and induction of IFNa, Mx1, PKR or TNFα gene expression at different times post infection were examined. The results showed on one hand, E1S with the highest ability to replicate, induce apoptosis and IFNa gene expression while VR-299 inhibited protein synthesis and induced Mx1 and PKR gene expression the most. The two Sp isolates induced the highest TNFα gene expression but differed in their ability to replicate, inhibit protein synthesis, and induce gene expression, with TA being more superior. Collectively, these findings point towards the adaptation by different virus isolates to suit environments and hosts that they patronize. Furthermore, the results also suggest that genetic identity is not prerequisite to functional similarities thus results of one aquabirnavirus isolate cannot necessarily be extrapolated to another.

## Introduction

Aquabirnaviruses are a genus in the family *Birnaviridae* that infect aquatic animals including fish, crustaceans and molluscs. They are non-enveloped, icosahedral double-stranded RNA viruses with genomes encoding two segments A and B [1]. Segment A comprises two overlapping open reading frames (ORFs), with the first (short) encoding a non-structural protein VP5 [2,3]. The second long ORF encodes structural proteins VP2 and VP3 as well as a non-structural protein VP4 [3–6]. Segment B has a single ORF that encodes the virus polymerase VP1 [7].

The type species of the genus aquabirnavirus is infectious pancreatic necrosis virus (IPNV) which is responsible for a highly contagious disease in salmonids. The genus has other members including Yellowtail Ascites Virus (YTAV), first isolated from yellowtail (*Seriola quinqueradiata*) in Japan [8] and Tellina virus (TV) [9] infecting other fish species. These viruses were tentatively called Marine birnaviruses (MABV) [10].

Aquabirnaviruses have high antigenic diversity and are classified into serogroups A and B. The former consists of nine serotypes designated A_1_ to A_9;_ also known as West Buxton (WB), Sp, Ab, He, Te, Canada (Can.) 1, Can. 2, Can. 3 and Jasper, respectively [11]. Serogroup B has only one serotype, Tv-1. In addition to this classification, the isolates have also been grouped into six geno-groups, giving good agreement with the serological classification [12]. Accordingly, sero-groups A_1_ and A_9_ correspond with geno-group 1; A_3_, A_2_ and A_4_ correspond with geno-groups 3, 5 and 6, respectively; A_7_ and A_8_ with geno-group 2; A_5_ and A_6_ with geno-group 4. Recently, a seventh group containing Japanese marine isolates obtained from fish and mulluscs has also been discovered [13].

The wide host range and antigenic diversity of aquabirnaviruses have been a source of confusion in terms of isolate nomenclature i.e. when a virus should be called IPNV and also to what extent findings of one isolate should be extrapolated to others. To harmonize the former, it was suggested that only isolates causing overt IPN in salmonids would be called IPNV [14]. All other isolates therefore were to be called aquabirnaviruses. This nomenclature however did not gain universal acceptance as some scientists continued making references to isolates infecting other species, for example E1S of eel, as IPNV [15]. Another problem associated with this nomenclature is that it did not take into account differences in virulence of identical isolates; a non-pathogenic isolate having an identical genetic sequence as a virulent one but differing only with two amino acids [16] would accordingly be excluded from list of IPNV. The purpose of the present study was to compare the mechanisms of cytopathogenesis between genetically and serologically identical and more distant isolates of aquabirnaviruses. Previous studies have shown contradictory findings related to protein shutdown and apoptosis in cells infected with IPNV [17–21], suggesting that pathogenic mechanisms are different. While infection profiles of different isolates of MABV and IPNV have been compared in different fish species and shown to differ in terms of mortalities induced in different species (i.e. in salmonids versus other fish species) [22,23], there is no documentation of the molecular basis of these differences. In this study, we specifically investigated the cell-pathogen interaction of IPNV isolates (TA & PT, serogroup A_2_, geno-group 5) and (VR-299, A_1_, geno-group 1) infecting salmonids on one hand and MABV (E1S, A3, geno-group 3) on the other. The results show differences in virus replication, protein synthesis inhibition, gene expression profiles and their ability to induce apoptosis.

## Materials and Methods

### Cells and Viruses

Rainbow trout gonad-2 (RTG-2) [24] and Chinook salmon embryonic (CHSE-214) cells [25] were grown in L-15 media with Glutamax (Gibco) supplemented with 10% and 5% FBS (Sigma Aldrich), respectively. Cells were cultured at 20°C until 90% confluence prior to virus infection.

### Virus propagation

Four virus isolates from different sero- and geno-groups were first propagated in RTG-2 cells in order to scale up the virus to be used in comparison experiments in this study. Two recombinant high and low virulent isolates previously made by reverse genetic [26] were chosen to represent the Sp serotype (A_2_). The high and low virulent isolates have the following virulence motifs T_217_A_221_ (TA) and P_217_T_221_ (PT), respectively in the VP2 capsid protein [16]. The E1S isolate (Ab serotype (A_3_)) originally from eel was provided by Professor Jen-Leih Wu (Academia Sinica, Taiwan). The VR-299 Isolate was kindly supplied by Professor Espen Rimstad (Norwegian University of Life Sciences). Table 1 lists virus isolates used in this study and the temperatures at which experiments were conducted in CHSE cells.

**Table 1:** Isolates used in the study.

Isolate	Source	Temperature	Genogroup	Serogroup	Reference
**E1S**	Isolated from eel	20°C	3	A3	[27]
**VR-299**	Isolated from rainbow trout	15°C	1	A1	[28]
**Sp (TA and PT)**	Template used isolated from Atlantic salmon	15°C	5	A2	[16,26]

### Virus infection and metabolic labeling

Six-well plates (Corning) containing approximately 90% confluent CHSE 214 were infected with MOI = 20 pfu/cell of each of the four isolates of IPNV. Infection was done in reverse order to result in the following time points 3, 12, 24 and 48 hours at the time of metabolic labeling. Metabolic labeling was done by washing the cells 3 times with PBS and incubation with Methionine-, Lysine- and L-glutamine-free Dulbecco’s modified Eagle’s medium (L-15 media, Sigma Aldrich) containing 20 μCi/ml S^35^ Methionine (Montebello), 1% L-glutamin (Sigma Aldrich) and 2% FBS for 30 minutes. After metabolic labeling, the cells were washed once with PBS, lysed using 250μl Cell M lysing reagent (Sigma Aldrich) and placed on a shaker for 15 minutes. The cells were then scraped to detach them and supernatants were collected in 1.5 ml eppendorf tubes followed by clearing by centrifugation at 11800 g for 5 minutes. Total protein in each lysate was estimated using Quick Start Bradford Protein Assay Kit (Biorad). To determine the amounts of labeled protein, equal amounts of total protein from each cell lysate was subjected to SDS-PAGE and blotted onto PVDF membrane. Finally, the membrane was incubated with a storage phosphor cassette overnight for autoradiography and the radioactivity was detected using the Typhoon (GE Health care). To quantify the levels of protein synthesis, ImageQuant software (GE Healthcare) was used to measure the density of a specific band common to all samples (infected and uninfected) at different time points post infection. The basis for selection of this band was that it had to be 1) prominent; 2) consistently expressed in infected and uninfected cells and 3) reflected or represented the general visual trend of protein expression of the samples. Host protein bands from infected cells and uninfected controls were expressed as percentages of infected relative to the uninfected controls.

### Virus Replication assays

To monitor the dynamics of virus replication during the infection period, confluent monolayers of CHSE-214 cells in 24-well plates (Corning) were infected with the different IPNV isolates at 20 pfu/cell. After incubation for 1 hour, virus supernatants were removed, the cells were washed once with PBS and maintenance media (L-15 media containing 2% FBS and 50μg/ml gentamycin) was added to the wells. At 3, 12, 24 and 48 hours post infection (hpi) supernatants were harvested and kept at 4°C until titration. In addition, infected cell monolayers were trypsinized by adding 100μl Trypsin EDTA (Sigma Aldrich) per well for about 5 min. Subsequently 100μl maintenance media was added and cells were subjected to 3 rounds of freeze-thawing to release the virus into the supernatant. Supernatants containing cell debris and virus were then transferred to eppendorf tubes, cleared by centrifugation and subsequently titrated. Eight samples of virus supernatants and five of supernatants containing virus released from cells, were titrated and used to infect confluent CHSE-214 grown in 96 wells plates. TCID_50_/ml was calculated using Karber’s method [29].

### Assessment of Apoptosis by flowcytometry

24-well plates containing confluent CHSE cells were used in a similar setup as described in the section above. In addition, parallel wells were treated with 1 μM Staurosporine for 12 hours as positive controls for apoptosis. At each sampling, the supernatant was transferred to a 2ml centrifugation tube or 5ml polystyrene round-bottomed tube (BD Biosciences). Adherent cells were washed twice with PBS prior to trypsinization. Trypsinization was done as described above for 5 minutes and stopped by adding fresh media. The trypsinized cells were then pooled with the original supernatants and cells were pelleted by centrifugation at 300 x g for 10 minutes. The supernatant was removed and cells were re-suspended in 100μl Hepes buffer containing 2μl Fluoresceine conjugated Annexin-V staining reagent (Annexin-V-FLUOS Staining Kit, Roche). After incubating for 30 minutes, the volume was adjusted to 200μl. To differentiate between apoptotic and necrotic cells, membrane permeability was assessed by adding Propidium Iodide (PI, Sigma Aldrich) to a final concentration of 8 μg/ml just before analysis. Flowcytometry was performed for 5,000 events using a Guava easyCyte Flow Cytometer (Millipore) while data analysis was performed using InCyte software, version 0.2 (Merck Millipore). The following parameters were measured to identify apoptotic cells: 1) the area pulse of forward light scatter (FSC-A) versus side scatter (SSC-A), and 2) fluorescent intensities of FITC (filter 525/30) and PI (filter 690/50) upon excitation with 20 mW 488 nm laser. Cell aggregates and debris were identified and excluded by using the width pulse of FSC-A versus area width of SSC-A.

### Quantitative real time PCR analysis

Real-time RT-qPCR was used to quantify the expression of IFNa, PKR, Mx1, and TNFα. Since no clear agreement on the nomenclature of fish IFN has been reached so far, it is noteworthy that the nomenclature used in this study was that suggested by Zou et al [30]. Cells were infected with the different isolates as described in the section on the protein shutdown with the exception that this time three parallels were used per treatment. At 3, 12, 24 and 48 hpi, cells were lysed using RLT buffer (RNeasy minikit, Qiagen) containing 10μl/ml β-mercaptoethanol. Total RNA was isolated by using the RNeasy Plus minikit according the manufacturer’s instructions and the concentration of RNA was determined by using the Nanodrop ND1000 (NanoDrop technologies). 400 ng of total RNA from each sample was used for cDNA synthesis, using a Transcriptor first strand cDNA synthesis kit (Roche) according to the manufacturer’s instructions. The cDNA was diluted five times and stored at -20°C until required.

Quantitative PCR was performed in 96 well plates using the LightCycler 480 system (Roche). For each reaction, 2μl cDNA was mixed with 10pmol gene specific primers and 10μl LightCycler 480 SYBR green I master mix. The final concentration was adjusted to 20μl using RNase free water. The sequences of primers used in the reactions are provided in Table 2. The cycling conditions for the PCR reactions were as follows: denaturation 94°C for 10 sec; annealing 60°C for 10 sec; elongation 72°C for 10 sec. The results were analyzed using the ΔΔCT relative quantification approach [31] with β-actin as a reference gene. Graphs were drawn with the help of GraphPad Prism 5.0 (GraphPad Software Inc.).

**Table 2:** Primers used for PCR.

Gene	Accession no.	Direction	Sequence
**B-actin**	AF012125	F	CCAGTCCTGCTCACTGAGGC
		R	GGTCTCAAACATGATCTGGGTCA
**Interferon α (IFNa)**	NM_001123570.1	F	TGGGAGGAGATATCACAAAGC
		R	TCCCAGGTGACAGATTTCAT
**Mx1 protein**	U66475	F	TGCAACCACAGAGGCTTTGAA
		R	GGCTTGGTCAGGATGCCTAAT
**Tumor necrosis factor α 2 (TNFα)**	DQ787158	F	AGATATTTAGGCGAACATTCAGTT
		R	TGACTCAGAGGAGTGGTACG
**PKR**	EF523422.1	F	TGAACACAGCCAGAAGAACAA
		R	GACTACCGCCACATAACTCCA

### Statistics

To compare the gene expression and virus titre results, two way ANOVA analysis followed by Bonferroni test to compare the difference between isolates at each time point was performed using GraphPad Prism 5.0 (GraphPad Software Inc.).

## Results

In the present study, an MOI = 20 pfu/cell was used. While this is high, it would guarantee that all cells were infected with more than 1 virus particle (using Poisson’s distribution), in principle ensuring a synconous 1-cycle infection kinetics. This was also in line with one of our recent publication [32].

When it comes to the temperatures used, E1S took 4 days to induce full CPE and to reach a virus titer of 10^7^ TCID50/ml at 15°C in CHSE cells in a previous study [33]. The same result was achieved in only 2 days at 20°C. For NVI-15 (TA isolate), it took 7 days to obtain full CPE and a virus titer of 10^7^ TCID50/ml in CHSE cells while at 20°C, the same number of days were required for CPE but only a virus concentration of 10^6^ TCID50/ml could be achieved. Against this background, cells in the present study were infected at temperatures optimal for respective virus propagation, namely 20°C for E-1S and 15°C for the others.

### Virus induced CPE

**Fig 1:**
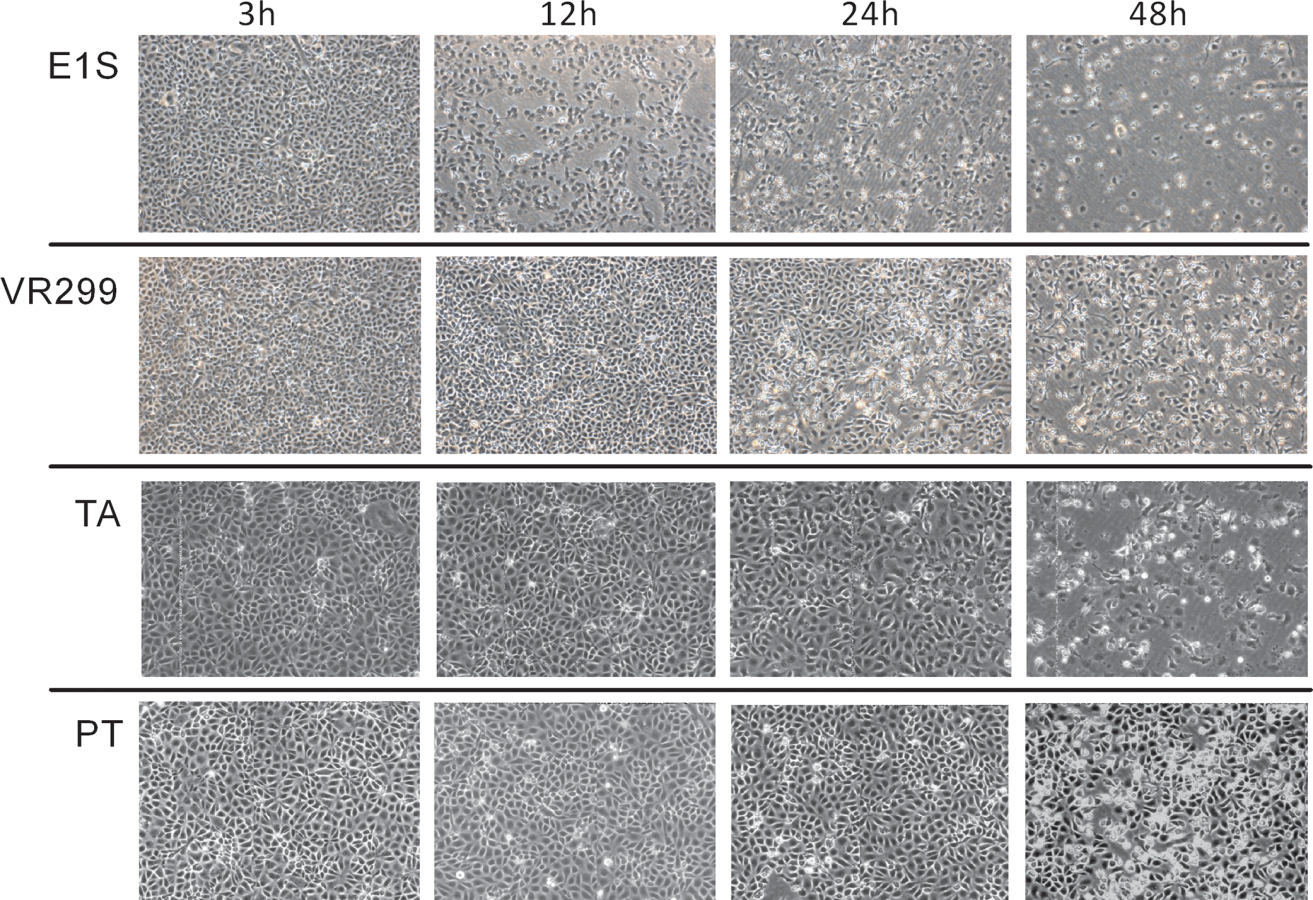
CPE development in CHSE-214 cells after infection with different aquabirnavirus isolates. Phase-contrast microscopical images at different times post infection.

As a starting point, the temporal difference between the isolates’ ability to induce CPE was assessed. All isolates induced CPE but at different time points following infection (Fig 1). E1S induced the earliest onset of CPE, seen at 12 hrs post infection (hpi), with rapid progression to 48 hpi. Onset of CPE by VR-299 and TA was at 24 hpi, however becoming only pronounced at 48 hpi, while the PT isolate showed the slowest induction, commencing at 48 hpi (Fig 1).

### Kinetics of virus titers in infected cells and supernatants

#### Intracellular virus loads

**Fig 2:**
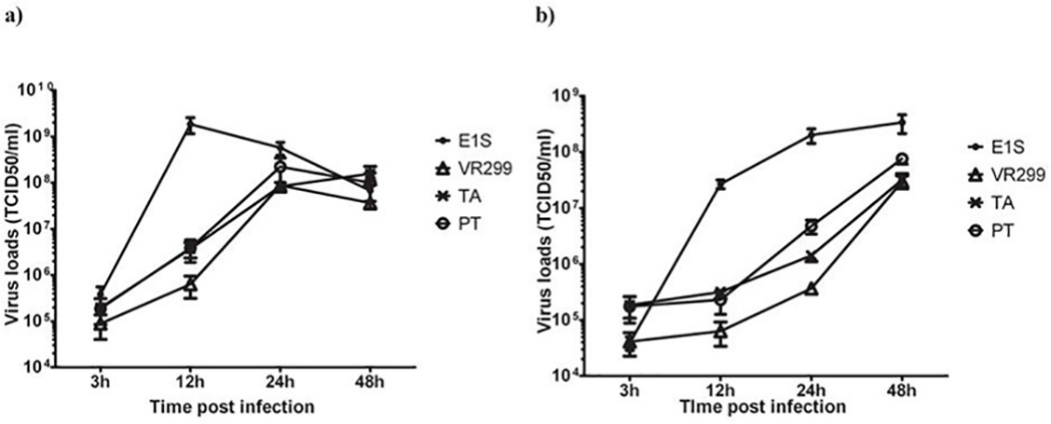
Replication curves for different aquabirnaviruses in CHSE-214 cells. Cell monolayers were infected with different IPNV isolates at 20pfu/cell. Amounts of virus in A) intracellular or B) supernatant at different time points post infections were determined by titration in CHSE-214 cells. The data are presented as mean ±S.E.M. log_10_ TCID50/ml of minimum of 5 replicated taken from at least two independent experiments.

Next, virus titers in the intracellular compartment were determined, starting at 3 hpi, all isolates were approximately the same level, but with E1S having a slightly higher intracellular titer than the others (Fig 2a; not statistically different). By 12 hpi, E1S had significantly higher titer (p<0.001) compared to other isolates, showing a 3.7 log_10_-fold increase, while for the other isolates, titer was 0.85 log_10_-fold to 1.3 log_10_ higher than at 3 hpi. By 24 hpi, E1S titers decreased by 0.7 log_10_ (compared to 12hpi), while for other strains there was an increase, about 1.76 log_10_, 1.36 log_10_ and 2.38 log_10_ for PT, TA and VR299, respectively. By 48 hpi, E1S showed a further decline of 0.9 log_10_ compared to 24 hpi, while VR-299 dropped by 0.6 log_10_ and PT by 0.5 log_10_. Only TA showed an increase, 0.3 log_10_ relative to the 24 hpi time point (Fig 2a).

#### Virus loads in supernatants

The kinetics of extracellular virus titers was different among the isolates and reflected intracellular events. E1S showed a 2.8 log_10_ increase from 3 to 12 hpi, while the other isolates ranged between 0.11 log_10_ and 0.43 log_10_ increase (Fig 2b). By 24 hpi, E1S leveled off (0.88 log_10_ increase) similar to VR-299 and TA over this time span (0.76 and 0.67 log_10_, respectively). For PT there was a 1.32 log_10_ increase. By 48 hpi, E1S remained at the same titer while the titre increase for the others ranged from about 1.2–1.91 log_10_, with the highest increase being for VR-299 (Fig 2b). In summary, E1S had generated about 60% of the total number of progeny over the incubation period by 24h post infection, while VR-299 produced the least at this time (below 2%). PT and TA on the other hand produced about 6 and 4% of the total progeny during the same period respectively. This means that VR-299, PT and TA generated more than 94% of the total virus particles over the last 24h of the incubation period (Fig 2b). On this basis, it can be assumed that the strategies explored by the virus to circumvent or counteract cellular antiviral responses would differ between the isolates.

### Inhibition of protein synthesis

**Fig 3:**
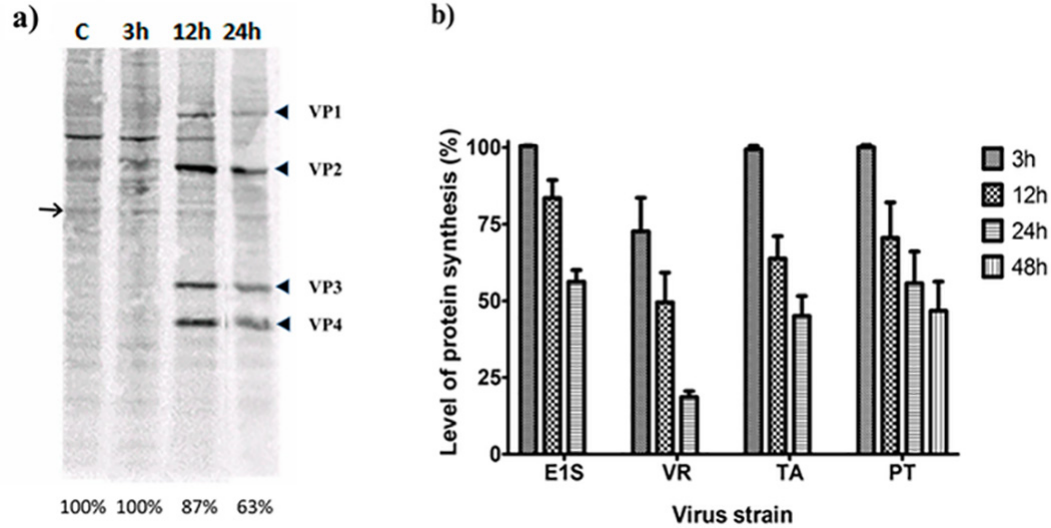
Impact of different aquabirnavirus isolates on global protein synthesis in CHSE-214 cells. Cells infected with either E1S, VR-299, TA or PT isolates collected at different times post infection were incubated with S35 Methionine for 30 min before lysis. Control cells were used in addition. Lysates (7μg total proteins) were subjected to SDS-PAGE, autoradiographed by incubation in storage phosphor cassettes before analysis by Typhoon. The ImageQuant software (GE Healthcare) was used to estimate the protein quantity by measuring the density of one band at different time points for each isolate. A) Autoradiograph image showing virus (VP1-4) and host protein (arrow) bands as an example. C = control sample at 48hrs post infection; Percentages = relative amounts of S35 Methionine-labeled host cell proteins calculated on the basis of the protein band shown as an arrow. B) Histogram of S35 Methionine-labeled host cell proteins of infected relative to uninfected cells as described above. Each entry represents three independent experiments and error bars are ± S.E.M. Key: VR = VR299.

We first analyzed the extent to which the different isolates induced shut-down of protein synthesis, a strategy used by many viruses to prevent cellular anti-viral responses. Two patterns of responses were observed. Cells infected with VR-299 and TA isolates showed a rapid onset with considerable reduction in protein synthesis (Fig 3a and 3b) by 24 hpi. In contrast, E1S and PT exhibited a slow and moderate onset. By 48 hpi, CPE was so extensive in all infected cells (except for PT isolate) that quantification of protein synthesis by labeling was not possible (thus not shown). Interestingly, as the host protein synthesis decreased, virus protein bands gained strength as can be seen from the strong intensity (Fig 3a), especially in cells infected with E1S and TA isolates (data not shown).

### Post-infection expression of selected genes

**Fig 4:**
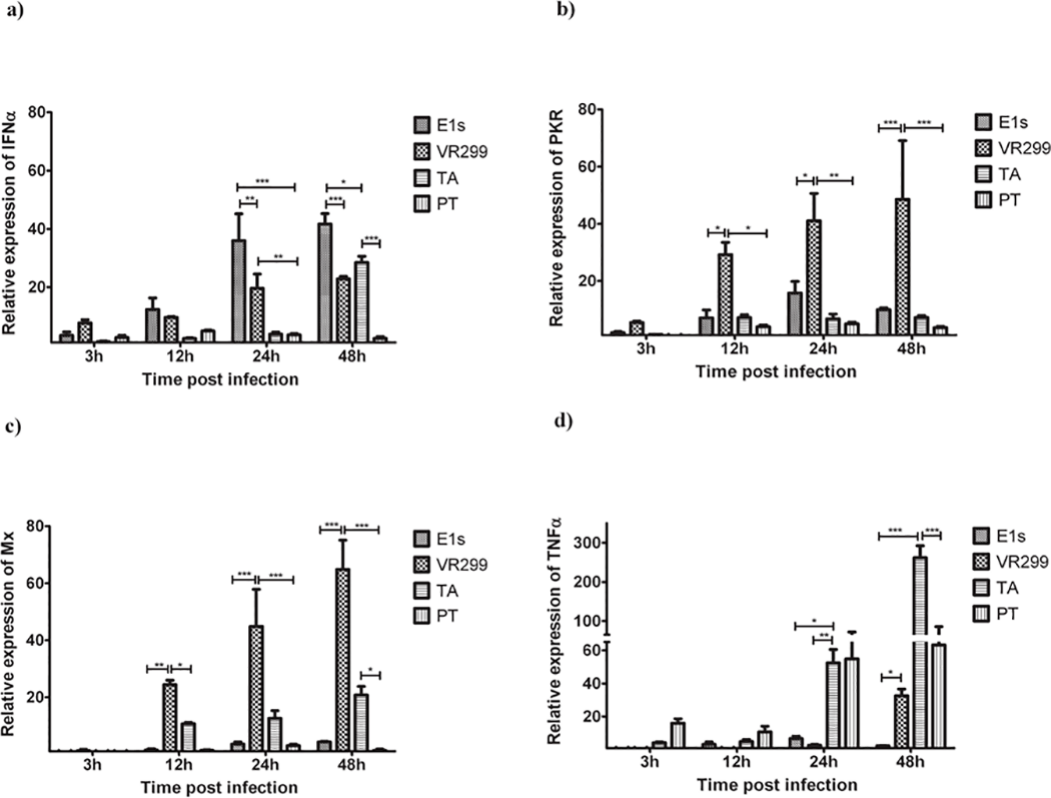
Type I IFN response induced by different isolates of aquabirnaviruses. Normalized gene expression of A) IFNα, B) PKR C) Mx1, and D) TNFα2 at different time points post infection ± S.E.M.. *P<0.05; **P<0.01; ***P<0.001. N = 3.

Selected genes associated with anti-viral responses were then analysed, IFNa, Mx1, and PKR, all by real-time PCR. E1S induced the highest increase in relative expression of IFNa from 12 to 24 hpi (Fig 4a), aligning with the highest production of viral progeny over this period. Interestingly, expression of Mx1 and PKR (Fig 4b and 4c) showed a very moderate increase over the same period, indicating that the virus inhibits down-stream induction of IFNa stimulated genes. VR-299 showed a less pronounced increase (25-fold up-regulation) of IFNa over the first 24h while the Mx1 and PKR induction were high already at 12 hpi (25-fold), increasing to more than 60-fold by 48 hpi (Fig 4b and 4c). The cellular responses to VR-299 were in strong contrast to that elicited by E1S, where the former replicated and produced more than 95% of its progeny under high expression of anti-viral factors. TA-infected cells showed slow onset of IFNa expression (5-fold at 24 hpi and 30-fold by 48h). Correspondingly, Mx1 and PKR also showed late up-regulation (20-fold at 48 hpi for Mx1 and less than 5-fold for PKR). This aligns with viral replication gaining momentum towards the end of the infection cycle and also with marked protein shut-down at 24 hpi (55% reduction). The ability of TA strains to replicate under high IFNa levels, has been shown before [34] and the fact that TA proliferates even when the cellular protein machinery is markedly lowered, would point towards the virus exploiting cap-independent initiation of translation.

For TNFα, the highest induction was after infection with TA and PT isolates (Fig 4d). E1S induced the weakest expression of TNFα while VR-299 only induced marked expression at 48 hpi.

### Flowcytometry results

**Fig 5:**
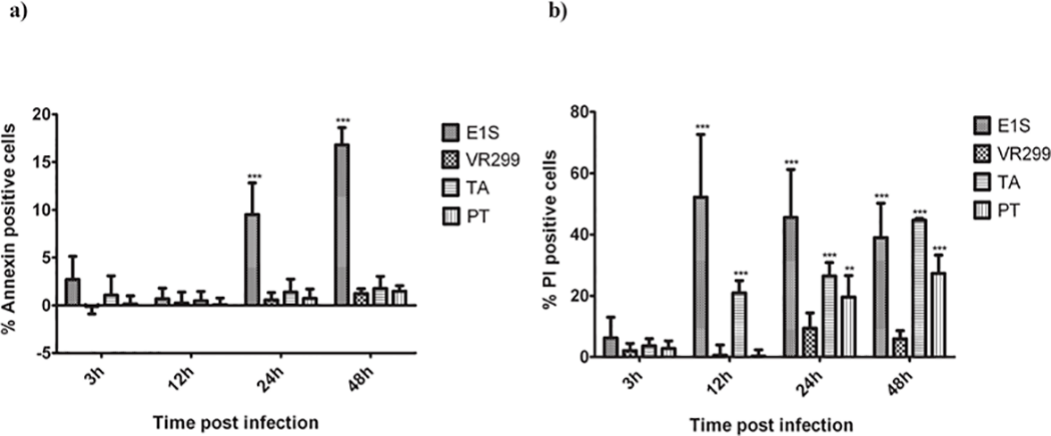
Cell death induced by different virus isolates of aquabirnaviruses. Flow cytometric analysis of FITC-Annexin (a) and PI (b) staining of cells infected with different isolates. The charts show the percentage of FITC-annexin stained cells (apoptosis) and PI stained cells (cells with compromised membranes) after subtracting the percentage of the FITC-annexin and PI positive percentages obtained from uninfected cells, respectively. The data represent the means of three independent experiments ±SD.

We used flowcytometry to assess the timing of apoptosis and necrosis induction in infected cultures. There was very little increase in Annexin V-positive (apoptotic cells) except for E1S where counts were significantly higher than other isolates at 24 and 48 hpi (Fig 5a). For the other virus strains, the percentage apoptotic cells remained below 5% throughout the study (Fig 5a).

PI positive or necrotic cells in the E1S infected cells peaked as early as 12 hpi, concomitant with high virus release to the supernatant (Figs 2a and 5b). For TA and PT, similar trends and correlates were seen; the highest percentage of PI-positive cells (Fig 5b) coincides with the highest increase in virus titer in the supernatant (Figs 2b and 5b). The most puzzling finding is for VR-299. While virus proliferation in the intracellular compartment starts late compared to the others, mirrored by a late release to the extracellular compartment, the majority of virus release is over the last 24h of the infection period (Fig 2a and 2b). However the PI-positive cells remain low throughout, peaks at 24 hpi but only 10% of the cells are positive (Fig 5b). It thus seems like virus is released without the cells going into dissolution, which makes it difficult to explain the mechanisms by which the virus is released to the extracellular compartment.

## Discussion

The main findings of this study can be summarized as follows: different aquabirnavirus isolates are associated with 1) differences in replication patterns over time; 2) consistent induction of IFNa relative to intracellular virus replication (apart from the PT strain); 3) differences in expression of anti-viral genes in infected cells after IFNa up-regulation. Furthermore, there was a remarkably low number of PI positive cells in VR-299 infected cells despite virus progeny being released to the supernatant.

E1S was propagated at a temperature (20°C) different from the other virus isolates (15°C), in line with optimal growth temperatures for the different strains. It is possible this influenced the findings since at higher temperatures, cells have higher metabolism and more rapid induction of immune responses, but for relevant comparison to be made, the viruses were propagated at their optimum temperatures. Nevertheless, the replication of aquabirnaviruses is sensitive to temperature and it is well established from previous studies that the replication of MABV and other isolates originally found at relatively high temperatures, is compromised at lower temperatures while the opposite is true for the cold water isolates [33,35–37]. A uniform temperature for all isolates would have therefore disadvantaged the replication/function of at least one of the isolates as previously demonstrated [33,37]. Temperature adaptation of aquabirnaviruses is likely one of the most prominent factors playing a role in determining the outcome of natural infections in different fish species. Similar findings have been reported for viruses like viral hemorrhagic septicemia virus [38]. Interestingly in this study, while E1S induced the highest IFNa gene expression, downstream responses like Mx1 and PKR were not induced suggesting that these isolates employ different strategies to evade host cell antiviral mechanisms.

CHSE cells were chosen to compare the different isolates in this study and this was done on the basis of 1) they were permissive to all isolates under study, 2) had previously been comparatively more extensively characterized than other salmonid cell lines, 3) availability. Although these cells can mount an innate response, it has been shown for IPNV that timing is important as further discussed below. The choice of cells is therefore unlikely to have contributed much to the outcome of this study. It is also noteworthy that CHSE cells themselves grow well at 20°C. At this temperature, the metabolism of the cells and likely the antivirus responses are higher than at 15°C. However, since some of the responses induced by E1S, e.g. Mx1 and PKR, were not more superior to other isolates, it is unlikely that temperature on cells per se plays an overarching effect on the growth of the viruses, rather, the different strains have adapted to the temperatures in their ecological niche.

E1S induced the quickest onset of CPE (Fig 1) coinciding with high virus multiplication in intracellular compartments and release to the supernatant compared to other isolates (Fig 2). In line with this, there was early induction of necrosis (Fig 5) while the fraction of apoptotic cells was highest at the end of the replication cycle. This finding contrasts a previous study [20] which showed the opposite that apoptosis precedes necrosis. The reason for these contrasting findings is not easily explained.

The antiviral effects of IFNa and Mx1 against several fish viruses including IPNV have been previously demonstrated [39–42]. Whether cells are protected or not is an issue of timing [39,41]. IFNa expression followed increase in intracellular virus levels, apart from E1S, where IFN up-regulation was delayed relative to increase in virus titer (Figs 2a and 4a). The down-stream responses to IFNa, i.e. induction of anti-viral genes (Mx1 and PKR) expression, was delayed and relatively down-played for all isolates except for VR-299 and the TA isolate, less pronounced for the latter. Despite the induction of antiviral genes, both VR-299 and TA produced progeny in intracellular compartments and with high release to the supernatant, i.e. more than 90% of virus released to the supernatant occurred between 24 and 48 hours post infection (Fig 2). The strategy of the PT isolate is puzzling; despite a vivid intracellular production of progeny between 12 and 48 hpi, there is literally no IFNa response and also no Mx1 or PKR responses seen in infected cells. Furthermore, CPE occurs late which coincides with PI-positive cells and release of virus to the supernatant. Obviously, the PT strain has an ability to evade the sensing mechanisms of the infected cell, which is surprising in light of the only difference between the TA and PT strains being 3 amino acid residues; P217T, T221A, and A247T [26]. The findings are in conformity with a previous report [42]. Further, the rapid induction of host cell protein shutdown, induction of CPE and increase in PI-positive cells by the TA isolate is probably a strategy for the virus to rapidly disperse from cell to cell prior to the onset of cellular, anti-viral mechanisms (Fig 4). These differences in mechanisms between identical isolates suggest that genetic identity is not prerequisite to functional similarities and therefore results of one isolate may not necessarily be extrapolated to others.

To overcome the drastic effect of IFN responses, many viruses employ different strategies to disrupt these responses. While some viruses disrupt Type I IFN induction, others antagonize the responses by disrupting the signaling cascade or, more specifically, target downstream genes and their products [43,44]. There are indications that aquatic birnaviruses, downplay IFN responses [42,45] but the underlying mechanisms remain obscure. In the present study, different IPNV isolates showed different abilities to induce IFNa or IFNa induced gene expression suggesting that different strategies are used by these isolates to overcome type I IFN responses. The weak ability of the Sp strains to induce IFNa and its downstream gene expression is remarkable and is in agreement with previous reports [42,45,46]. While the PT isolate suppressed the induction of these genes throughout the observation period, the TA isolate only delayed the induction until 48 hpi. The reason behind these findings is not clear although it is not unlikely that IFN responses may be used for the benefit of the virus, for example in the induction of necrosis/apoptosis and virus release. These findings bring to the fore the deficit in understanding the interplay between aquabirnaviruses and the IFN system and additional studies are required to understand the underlying strategies.

The percentage of PI-positive cells for the TA and E1S strains are in strong contrast to what was observed for VR-299, where they never exceeded 10% (Fig 5b). No doubt, the release of virus to the supernatant was delayed compared to the other strains (Fig 2b), but still the release of virus did not correlate with PI-positivity, particularly at 48 hpi. This contradiction is difficult to explain. One possibility is that VR299 is very efficient at inducing necrosis [19] so that lysed cells are discarded as debris when preparing cells for flow cytometry analysis. The possibility of an alternative release-mechanism has also to be considered but the general understanding is that IPN virus is released through induction of necrosis in the infected cell [19].

The differences in infection kinetics of the different isolates agree well with mortalities (or lack of) observed in previous studies where IPNV or MABV were used to infect salmonids or other species [22,23,47]. Collectively, these findings support the view that a distinction should be made between IPNV and MABV, where only isolates causing overt disease in salmonids should be referred to as IPNV [14] and vice-versa.

Contradicting reports of protein shutdown by IPNV in RTG-2 cells have previously been observed, with some suggesting induction [18] and others the lack of it [17]. Although CHSE cells were used in the present study instead of RTG-2 cells (in previous studies), the findings here in general support reports [18,32,42] that propose that different isolates of aquabirnaviruses induce different onsets/rates of host protein shut down. These results are however at variance with the findings of others [17] who found no shut down. While the same isolate VR299 was used in both studies, the results of the present study show that this virus induced the quickest and most pronounced protein shutdown. A possible explanation of the contradiction between these results is most likely methodological.

TNFα plays different roles with respect to viral infection. It may either inhibit or promote virus replication in infected cells [48,49]. In the current study, different isolates induced different levels of TNFα expression. Induction of TNFα expression by the E1S isolate has previously been studied using zebra fish embryonic cell line where it was suggested that it is involved in regulation of cell death [15,50]. In that study, the induction of TNFα started as early as 6 hpi and was detected up to 24 hpi, in contrast to results of the present study (Fig 4d). This discrepancy is likely due to different host cells used suggesting that differences in host cells should be taken into account when drawing conclusions about host-virus interaction. Following extensive cell death proceeding infection, most of the remaining cells become persistently infected but survive, forming a monolayer. It is therefore not unlikely that TNFα can promote cell survival and also play a role in virus persistence. The fact that PT in this study showed the least ability to induce CPE as well as the highest ability to induce TNFα at early time points (3 and 12 hpi, Fig 4d) supports this argument. Nevertheless, further studies are needed to clarify this.

Apoptosis refers to programmed cell death and is sometimes elevated during virus infections. It may also be induced by viruses to facilitate their release from cells [51], while in other cases it is induced as an attempt by the cells to commit suicide and therefore arrest the replication and spread of viruses [52]. Induction of apoptosis following infection with aquabirnaviruses has been a subject of several studies with conflicting results [19–21]. The findings of the present study demonstrate that only E1S induced significant apoptosis (p<0.001) (Fig 5a) while for the other strains, the number of apoptotic cells remained at a minimum.

In conclusion, we show that different isolates of aquabirnaviruses induce different patterns of host responses. These differences may be explained by adaptation to a wide variety of hosts.
